# Goal-directed transfusion protocol via thrombelastography in patients with abdominal trauma: a retrospective study

**DOI:** 10.1186/1749-7922-9-28

**Published:** 2014-04-15

**Authors:** Jianyi Yin, Zhenguo Zhao, Yousheng Li, Jian Wang, Danhua Yao, Shaoyi Zhang, Wenkui Yu, Ning Li, Jieshou Li

**Affiliations:** 1 Department of Surgery, Jinling Hospital, Nanjing University School of Medicine, Nanjing, China; 2 Department of General Surgery, The Affiliated Jiangyin Hospital of Southeast University Medical College, Jiangyin, China

**Keywords:** Transfusion, Thrombelastography, Trauma-induced coagulopathy, Abdominal trauma

## Abstract

**Introduction:**

The optimal transfusion protocol remains unknown in the trauma setting. This retrospective cohort study aimed to determine if goal-directed transfusion protocol based on standard thrombelastography (TEG) is feasible and beneficial in patients with abdominal trauma.

**Methods:**

Sixty adult patients with abdominal trauma who received 2 or more units of red blood cell transfusion within 24 hours of admission were studied. Patients managed with goal-directed transfusion protocol via TEG (goal-directed group) were compared to patients admitted before utilization of the protocol (control group).

**Results:**

There were 29 patients in the goal-directed group and 31 in the control group. Baseline parameters were similar except for higher admission systolic blood pressure in the goal-directed group than the control group (121.8 ± 23.1 mmHg vs 102.7 ± 26.5 mmHg, p < 0.01). At 24 h, patients in the goal-directed group had shorter aPTT compared to patients in the control group (39.2 ± 16.3 s vs 58.6 ± 36.6 s, p = 0.044). Administration of total blood products at 24 h appeared to be fewer in the goal-directed group than the control group (10.2 [7.0-43.1]U vs 14.8 [8.3-37.6]U, p = 0.28), but this was not statistically significant. Subgroup analysis including patients with ISS ≥16 showed that patients in the goal-directed group had significantly fewer consumption of total blood products than patients in the control group (7[6.1, 47.0]U vs 37.6[14.5, 89.9]U, p = 0.015). No differences were found in mortality at 28d, length of stay in intensive care unit and hospital between the two groups.

**Conclusions:**

Goal-directed transfusion protocol via standard TEG was achievable in patients with abdominal trauma. The novel protocol, compared to conventional transfusion management, has the potential to decrease blood product utilization and prevent exacerbation of coagulation function.

## Introduction

Uncontrollable hemorrhage is a major cause of early death in trauma patients [1]. Hemorrhage may occur due to direct injury, and is frequently complicated by coagulopathy [2,3]. Post-injury coagulopathy may exacerbate hemorrhage and contribute to poor outcome and an increased transfusion requirement [4,5].

Blood transfusion is an essential component in trauma management. The goal of transfusion includes improvement of tissue oxygen delivery by replacing red blood cell, as well as prevention and correction of coagulation dysfunction by supplementing appropriate blood components. However, the optimal transfusion protocol for trauma patients remains unknown. In lack of guidance by rapid and comprehensive tools monitoring coagulation status, current transfusion protocols are unable to utilize blood products according to individual demands. As a consequence, these protocols are likely to lead to inappropriate and excessive administration of blood products, which is associated with increased burden of blood product supply and risk of transfusion-related morbidity.

In recent years, viscoelastic hemostatic assays (VHA), including thrombelastography (TEG) and thrombelastometry, have been demonstrated to be ideal methods of monitoring coagulation function in trauma patients [6,7]. Furthermore, several studies have suggested the potential of VHA tests to guide component blood transfusion in a variety of patient groups [8-12]. In particular, a recent study by Kashuk et al. [13] showed that goal-directed transfusion based on rapid TEG was useful in managing trauma-induced coagulopathy, with the potential to reduce blood product administration in trauma patients.

A goal-directed transfusion protocol via TEG was implemented in our department since 2010 [14]. In the present study, we assessed the utilization of the protocol in abdominal trauma management by comparing outcomes of patients admitted before and after implementation of the protocol. We aimed to determine if the novel transfusion protocol could be successfully integrated in abdominal trauma management, and identify potential benefits of the protocol compared to conventional transfusion management.

## Materials and methods

This cohort study analyzed the prospectively collected data of patients with abdominal trauma at Department of General Surgery, Jinling Hospital. The study was approved by the ethics committee of Jinling Hospital. Waiver of informed consent from patients was approved because of the observational nature of the study. Jinling Hospital is a tertiary teaching hospital in Nanjing, China. The Department of General Surgery is responsible for medical and surgical care of patients with abdominal trauma admitted to the emergency department (ED) of the hospital. At ED, a consulting surgeon judges the need for emergency laparotomy of the abdominal trauma patient. The patient is subsequently transferred to one of the two surgical intensive care units (SICU) of our department from ED if emergency laparotomy is not needed, or from operation room after emergency laparotomy. Non-operative care is provided by a team of surgeons and SICU specialists following previously published guidelines [15].

### Study population

We searched the abdominal trauma database to identify potential patients between November 2008 and October 2012. Inclusion criteria were age older than 18 years, abdominal abbreviated injury scale ≥2, and requirement of 2 or more units of red blood cell (RBC) transfusion within 24 hours of ED admission. Exclusion criteria included time interval between injury and ED admission >24 hours, major traumatic brain injury (head abbreviated injury scale ≥3), end-staged liver disease, pregnancy, and history of anti-coagulation therapy in the latest 3 months.

All included patients were subsequently divided into 2 groups according to the time of admission. Patients between November 2008 and October 2010, who received conventional transfusion management, were assigned to the control group, whereas patients between November 2010 and October 2012, who were managed with the goal-directed transfusion protocol, were assigned to the goal-directed group.

### Transfusion protocol

At ED, patients with abdominal trauma might receive preemptive transfusion of 2 units of RBC and 2–4 units of fresh frozen plasma (FFP) following initial fluid resuscitation when hemoglobin level was below 90 g/L or showed active bleeding signs. Once the patient was planned to be transferred to our department, subsequent transfusion decisions were made by the treating surgeon or SICU specialist.

Patients in the control group received conventional transfusion management, which was based on individual experience and interpretation of conventional coagulation testing results of the treating surgeon or SICU specialist. RBC and FFP were delivered at a ratio of 1:1–1:2. Platelet and cryoprecipitate were administrated in selected cases.

The TEG 5000 thrombelastograph hemostasis analyzer system (Haemoscope Corporation, Niles, USA) was initially introduced to our department for monitoring post-operative coagulation function. The device enables point-of-care coagulation assay of whole blood at the patient’s temperature. The device undergoes quality control daily according to the manufacturer’s instructions. TEG analysis is carried out within 4 minutes of blood sample collection. The whole blood sample is placed in a manufacturer-supplied vial containing kaolin, and 0.35 ml of the blood sample is added to a cup, followed by adjustment of the temperature setting to the patient’s temperature. TEG assay is then started and stopped when reaching full tracing. A number of parameters are generated from the TEG tracing, each representing an aspect of hemostasis. The R value is the time from the beginning to the onset of clot formation, representing the activity of enzymatic clotting factors. The α angle is the angle between the tangent line and the horizontal line of the tracing, representing the activity of fibrinogen. The maximal amplitude (MA) is the overall clot strength, indicating the platelet activity.

**Figure 1:**
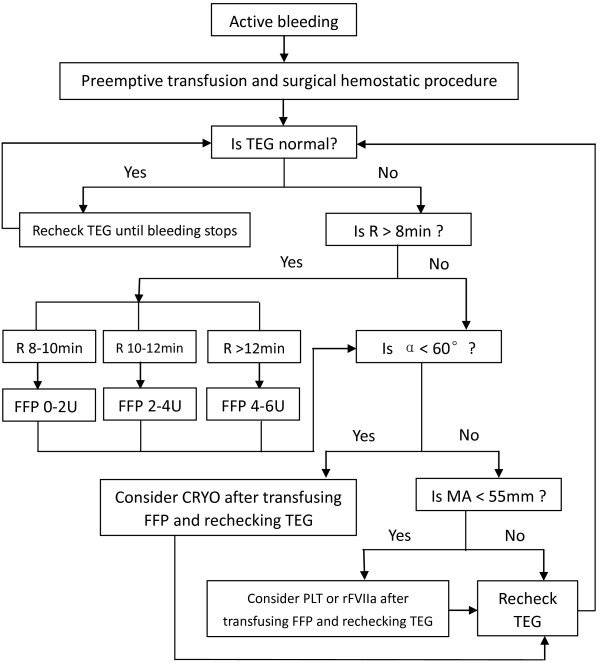
Goal-directed transfusion protocol via TEG.

Patients in the goal-directed group were managed with goal-directed transfusion protocol based on TEG results (Figure 1). The protocol was developed by a group of surgeons, SICU specialists, and transfusion specialists, and was introduced to all surgeons and SICU specialists of our department before its implementation in November 2010. The algorithm of the protocol was shown as hard copies in SICU, and two attending surgeons and two SICU specialists ensured utilization of the protocol as the leaders of abdominal trauma management. In specific, standard TEG test was ordered by the treating surgeon or SICU specialist when the patient with abdominal trauma was admitted to SICU, or had active bleeding at ED, operation room (OR), or SICU. Whole blood sample was transferred immediately to the SICU of our department, where it was analyzed. Results were fed back via in-hospital communication system to the treating surgeon or SICU specialist, who determined further transfusion management according to the goal-directed transfusion protocol. The goal-directed transfusion might occur at ED, OR, or SICU. Subsequent TEG tests were ordered until the patient had no active bleeding or coagulopathy.

### Data collection

Data of all included patients from ED, SICU, OR, blood bank, and laboratory were linked. Demographic characteristics (age and gender), injury severity indices (injury mechanism, injured organs, injury severity score [ISS], abdominal abbreviated injury scale [AIS]) were collected. Administration of component blood products within 24 hours of ED admission was also recorded. Clinical and laboratory parameters of interest included vital signs (body temperature, heart rate, and systolic blood pressure), arterial blood gas results (pH, lactate, and base excess), blood cell counts (hemoglobin concentration, RBC count, and platelet count), albumin and calcium concentration, international normalized ratio (INR) and activated partial thromboplastin time (aPTT) at ED admission and 24 h. TEG parameters (R value, α angle, and MA value) of the first TEG test and the follow-up one between 24–48 hours after the first one were collected from patients in the goal-directed group. In addition, mortality at 28d, length of stay in ICU and hospital were noted. An independent investigator checked the accuracy of collected data before analysis.

### Statistical analysis

We used SPSS software (v19.0 for Windows, IBM Corporation, USA) for statistical analysis. Normality of distribution was analyzed by Kolmogorov-Smirnov test. Continuous variables with normal distribution and skewed distribution were analyzed using Student’s t test and Mann–Whitney u test, respectively. Categorical variables were analyzed using chi-square test. Significance was considered as *p* < 0.05.

## Results

### Patient characteristics

**Figure 2:**
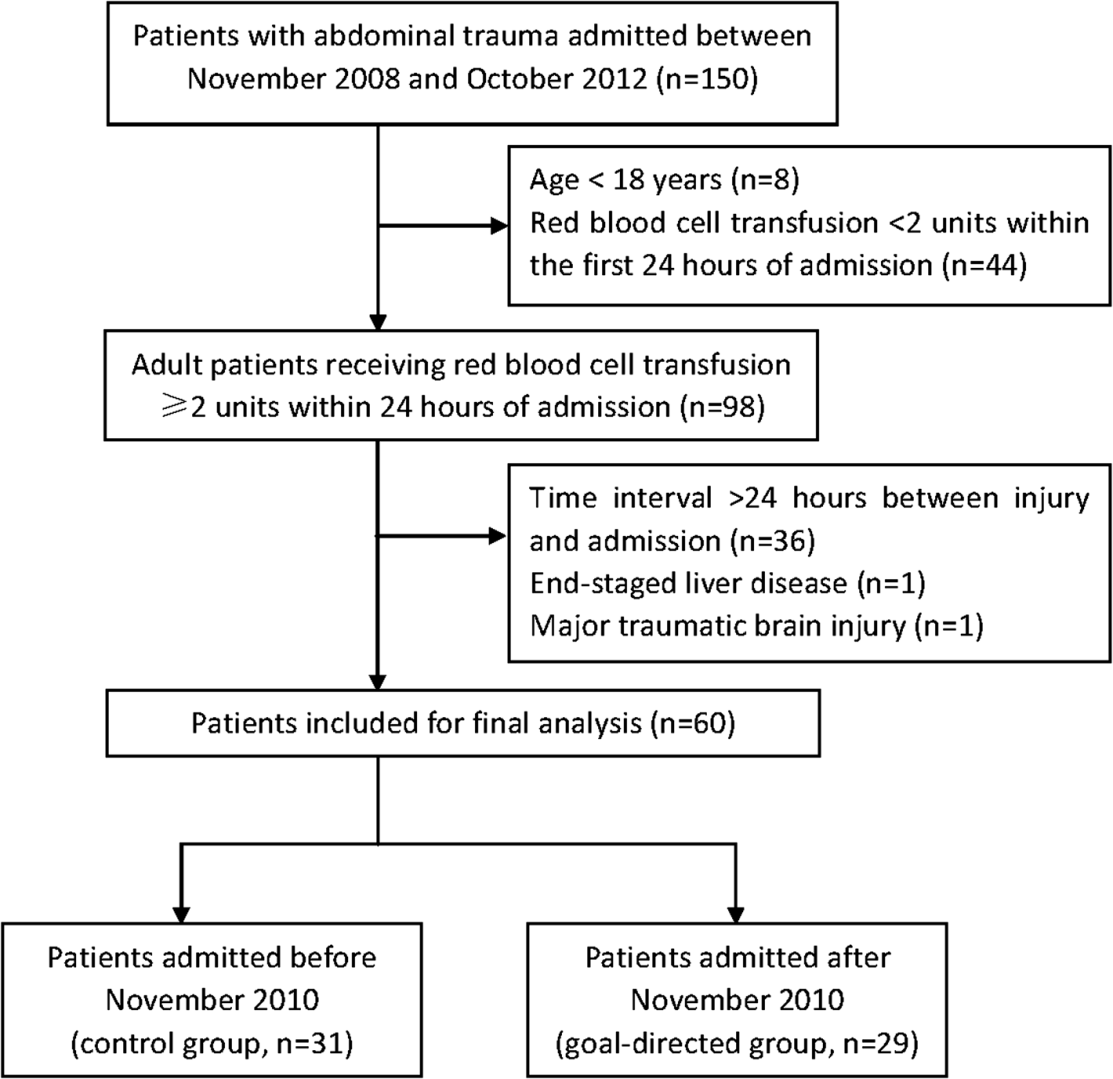
Flowchart showing patient inclusion and exclusion.

A total of 150 patients with abdominal trauma were admitted between November 2008 and October 2012, of whom 98 met the inclusion criteria. Thirty-eight patients were excluded due to prolonged time interval between injury and ED admission (n = 36), end-staged liver disease (n = 1), and major traumatic brain injury (n = 1), leaving 60 patients for final analysis (Figure 2).

There were 31 patients in the control group and 29 in the goal-directed group. The two groups were comparable in terms of age and gender. The control group and the goal-directed group had similar ISS (14.3 ± 5.7 vs 16.2 ± 8.0, p = 0.28) and abdominal AIS (3.1 ± 0.7 vs 3.1 ± 0.9, p = 0.86). There were, however, more frequent patients with pancreatic injury in the goal-directed group than the control group (44.8% vs 16.1%, p = 0.015). All but 3 patients (2 in the control group and 1 in the goal-directed group) underwent emergency operation for control of intra-abdominal bleeding or repair of intra-abdominal organ injury (Table 1).

**Table 1:** **Patient characteristics**^**a**^

	**Overall (n = 60)**	**Control group (n = 31)**	**Goal-directed group (n = 29)**	**p**
Age (year)	41.7 ± 14.2	42.8 ± 15.6	40.5 ± 12.8	0.53
Gender				
Male	49(81.7)	26(83.9)	23(79.3)	0.65
Female	11(18.3)	5(16.1)	6(20.7)	
Mechanism of injury				
Blunt	50(83.3)	27(87.1)	23(79.3)	0.64
Penetrating	10(16.7)	4(12.9)	6(20.7)	
ISS	15.2 ± 6.9	14.3 ± 5.7	16.2 ± 8.0	0.28
Abdominal AIS	3.1 ± 0.8	3.1 ± 0.7	3.1 ± 0.9	0.86^b^
Involved abdominal organ				
Spleen	24(40.0)	15(48.4)	9(31.0)	0.17
Liver	14(23.3)	9(29.0)	5(17.2)	0.28
Pancreas	18(30.0)	5(16.1)	13(44.8)	0.015
Vessel	5(8.3)	4(12.9)	1(3.4)	0.39
Stomach	4(6.7)	1(3.2)	3(10.3)	0.35
Duodenum	6(10.0)	4(12.9)	2(6.9)	0.73
Intestine	12(20)	5(16.1)	7(24.1)	0.44
Colon	14(23.3)	6(19.4)	8(27.6)	0.45
Rectum	2(3.3)	1(3.2)	1(3.4)	1.00
Emergency operation	57(95)	29(93.5)	28(96.6)	1.00
ICU stay (day)	10.1 ± 9.2	8.1 ± 5.5	12.2 ± 11.8	0.28^b^
Hospital stay (day)	13.4 ± 10.0	11.3 ± 6.2	15.6 ± 12.7	0.10
Mortality at 28d	5(8.3)	2(6.5)	3(10.3)	0.94

^a^Data are presented as mean ± SD or number(%).

^b^Mann–Whitney u test.

ISS, injury severity score; AIS, abbreviated injury scale, ICU: intensive care unit.

### Administration of blood products

Administration of blood products within 24 hours of ED admission was presented in Table 2. No significant differences were found in the proportion of patients receiving FFP (100% vs 96.8%, p = 1.0), platelet (13.8% vs 29.0%, p = 0.15), and cryoprecipitate (24.1% vs 29.0%, p = 0.67) between the goal-directed group and the control group. Administration of RBC, FFP, platelet, cryoprecipitate, and total blood products was fewer in the goal-directed group than the control group, but this did not reach statistical significance. We further performed subgroup analysis including patients with ISS ≥16. The results showed that patients in the goal-directed group (n = 16) had significantly fewer consumption of RBC (4[3,11.5]U vs 14[7.5, 32]U, p < 0.01), FFP (4[2.9, 9.8]U vs 10.5[5.6, 15.7]U, p = 0.036) and total blood products (7[6.1, 47.0]U vs 37.6[14.5, 89.9]U, p = 0.015) than patients in the control group (n = 13), whereas consumption of platelet and cryoprecipitate was not significantly different. Furthermore, the cost of total blood product appeared to be lower in the goal-directed group than the control group ($227.5[152.9, 1221.7] vs $329.0 [197.2, 2904.8]), but this was not significantly different (p = 0.156).

**Table 2:** **Administration of blood products at 24 h**^**a**^

	**Control group (n = 31)**			**Goal-directed group (n = 29)**			**p**
	**Number**	**Median**	**IQR**	**Number**	**Median**	**IQR**	
RBC (U)	31	6.5	4-14	29	5	3-13	0.22
FFP (U)	30	6.1	4-10.7	29	5.7	3.4-10	0.54
PLT (U)	9	0	0-10	4	0	0-0	0.15
CRYO (U)	9	0	0-10	7	0	0-5	0.68
Total (U)	31	14.8	8.3-37.6	29	10.2	7.0-43.1	0.28

^a^Data were analyzed using Mann–Whitney u test.

RBC: red blood cell; FFP: fresh frozen plasma; PLT: platelet; CRYO: cryoprecipitate; IQR: interquartile range.

### Clinical and laboratory parameters

Clinical and laboratory parameters of interest at ED admission and 24 h were summarized in Table 3. Patients in the goal-directed group had significantly higher systolic blood pressure at ED admission (121.8 ± 23.1 mmHg vs 102.7 ± 26.5 mmHg, p = 0.005) and lower pH (7.39 ± 0.06 vs 7.41 ± 0.04, p = 0.048) at 24 h than patients in the control group. In addition, aPTT at 24 h was significantly shorter in the goal-directed group compared to the control group (39.2 ± 16.3 s vs 58.6 ± 36.6 s, p = 0.044), while admission aPTT was similar (25.7 ± 4.8 s vs 28.4 ± 6.4 s, p = 0.09). No significant differences were observed in other parameters between the two groups.

**Table 3:** Clinical and laboratory parameters

	**At ED admission**					**At 24 h**				
	**Control group (n = 31)**		**Goal-directed group (n = 29)**		**p**	**Control group (n = 31)**		**Goal-directed group (n = 28)**		**p**
	**Number**	**Mean ± SD**	**Number**	**Mean ± SD**		**Number**	**Mean ± SD**	**Number**	**Mean ± SD**	
Temperature (°C)	31	36.4 ± 0.3	29	36.4 ± 0.3	0.98	31	37.2 ± 0.7	28	37.2 ± 0.6	0.84
HR (/min)	31	100.3 ± 19.5	28	91.8 ± 18.7	0.09	31	101.4 ± 18.6	28	96.9 ± 18.3	0.35
SBP (mmHg)	31	102.7 ± 26.5	28	121.8 ± 23.1	0.005	31	122.4 ± 16.8	28	122.6 ± 14.7	0.97
Hb (g/L)	30	121.1 ± 20.6	28	122.5 ± 24.0	0.82	31	105.5 ± 15.2	27	106.6 ± 18.9	0.8
RBC (×10^12^/L)	30	3.9 ± 0.6	27	4.1 ± 0.7	0.27	31	3.4 ± 0.5	27	3.5 ± 0.6	0.69
PLT (×10^9^/L)	30	186.2 ± 52.9	28	181.1 ± 59.0	0.73	31	113.0 ± 45.1	27	116.6 ± 47.7	0.77
pH	16	7.38 ± 0.05	14	7.38 ± 0.04	0.66	25	7.41 ± 0.04	27	7.39 ± 0.06	0.048
Lactate (mmol/L)	16	2.8 ± 1.5	14	3.1 ± 2.4	0.68	25	2.6 ± 1.7	27	2.1 ± 1.4	0.18^a^
BE (mmol/L)	16	(-3.9) ± 3.4	14	(-3.0) ± 3.5	0.48	25	(-2.7) ± 4.6	27	(-2.4) ± 2.5	0.75
Albumin (g/L)	28	38.3 ± 6.1	28	38.1 ± 7.3	0.92	31	33.2 ± 5.8	27	33.6 ± 4.5	0.79
Calcium (mmol/L)	25	2.1 ± 0.2	27	2.1 ± 0.2	0.91	31	2.0 ± 0.2	27	2.0 ± 0.2	0.28
INR	27	1.1 ± 0.2	28	1.1 ± 0.1	0.73	26	1.2 ± 0.2	24	1.2 ± 0.2	0.97
aPTT (s)	27	28.4 ± 6.4	28	25.7 ± 4.8	0.09	26	58.6 ± 36.6	24	39.2 ± 16.3	0.044^a^

^a^Mann-Whitney u test.

The first TEG test in the goal-directed group showed R value of 10.1 ± 4.7 min, α angle of 44.1 ± 16.1, and MA value of 50.0 ± 12.1. A follow-up TEG test between 24–48 hours after the first TEG test was available from 21 patients, with improved R value of 8.5 ± 4.7 min (p = 0.037), α angle of 51.1 ± 11.5 (p < 0.001), and MA value of 52.0 ± 13.3 (p = 0.11).

### Clinical outcomes

There were 3 deaths (1 for exsanguination at 24 h, 1 for multiple organ dysfunction at 72 h, 1 for coagulopathy at 14d) in the goal-directed group and 2 deaths for coagulopathy (1 at 48 h and 1 at 72 h) in the control group. No significant differences were found in mortality at 28d, length of stay in ICU and hospital between the two groups.

## Discussion

This cohort study showed that goal-directed transfusion protocol via TEG was applicable in patients with abdominal trauma, and was associated with a trend towards fewer blood product utilization and better coagulation profile at 24 h compared to conventional transfusion management. The results support the use of TEG in guiding transfusion management in patients with abdominal trauma.

First, this study provides supplemental evidence for using TEG to guide transfusion management in the trauma setting. TEG has been shown to be helpful in detecting post-injury coagulopathy and directing transfusion management in patients with severe multiple trauma [13], but the use of TEG in patients with lower injury severity has not been thoroughly investigated, which may be due to the relatively low incidence of coagulopathy in moderately injured patients [2]. In this study, the majority of included patients sustained moderate abdominal injury, as suggested by mean ISS of 15.2 and mean abdominal AIS of 3.1. Despite the relatively low injury severity, our patients were still exposed to risk of coagulation dysfunction, as suggested by aggravation of INR and aPTT during the first 24 hours of ED admission. The exacerbation of coagulation function might be associated with primary injury, second hit of operation, blood loss, and massive fluid resuscitation [16]. We found that TEG had reasonable value in monitoring coagulation function in these patients, and goal-directed transfusion protocol via TEG could be successfully implemented.

Second, our results showed that goal-directed transfusion protocol via TEG had the potential to reduce administration of component blood products. Although not statistically significant, patients managed with goal-directed transfusion protocol received fewer component blood products, especially RBC and FFP than patients receiving conventional transfusion management. In subgroup analysis including patients with ISS ≥ 16, we showed that goal-directed transfusion protocol led to significant reduction in administration of RBC, FFP, and total blood products. These results are consistent with the findings of several previous studies [8,11,13]. Moreover, we found that the reduction in blood product administration did not compromise perfusion status and oxygen delivery capacity, as evidenced by similar lactate level, hemoglobin concentration, and RBC count at 24 h between the two patient groups. The reduction of blood product administration is important in two aspects. First, it relieves the burden of blood product supply, and may have the potential to decrease the cost of blood products for patients. Second, it is likely to lower transfusion-related morbidity, such as coagulopathy, transfusion-related acute lung injury, and infection [17]. However, these findings must be interpreted with caution given the small sample size of the study and subgroup analysis.

Third, goal-directed transfusion protocol appears to be better than conventional transfusion management in preventing coagulation function exacerbation after transfusion. In recent years, there is improving understanding in acute traumatic coagulopathy (ATC), which is resulted from tissue injury and hypoperfusion due to trauma. Subsequent medical interventions, such as massive transfusion, may further exacerbate coagulation dysfunction and lead to trauma-induced coagulopathy (TIC) [18]. In this study, we observed that patients in the goal-directed group had better coagulation profile at 24 h, as indicated by shorter aPTT, than patients in the control group. Furthermore, the TEG parameters were significantly improved in patients managed with goal-directed transfusion protocol. There are two possible explanations for these findings. First, goal-directed transfusion protocol could prevent coagulation function worsening through supplementing appropriate blood component according to individual requirement. Second, the reduction of blood product utilization, as a result of the use of goal-directed transfusion protocol, might lower the risk of TIC secondary to massive transfusion. However, these findings needed to be interpreted carefully, since aPTT can represent only part of the coagulation system, and is affected by multiple factors [19]. Moreover, although aPTT results were available in more than 83.3% and follow-up TEG results were available in 72.4% of patients, missing data might reduce the power of the results.

The present study did not observe benefits of goal-directed transfusion protocol on mortality, which is not surprising because of the small sample size and moderate injury severity in overall. The previous study by Kashuk et al. [13] did not conclude the effect of goal-directed transfusion management on mortality either, because of incomparable injury severity between the patient groups. Considering the potential of goal-directed transfusion protocol in decreasing transfusion-related morbidity and correcting post-injury coagulopathy, it would be justified to infer that goal-directed transfusion protocol might improve mortality of trauma patients. Further studies are needed to investigate this issue.

Several limitations are worth considering when interpreting the results of this study. First, this is a retrospective study with small sample size. Due to the retrospective nature, we could not achieve two identical patient groups, as manifested by different admission systolic blood pressure between the two groups. Second, we did not abandon conventional coagulation tests after implementation of TEG. Therefore, the influence of conventional coagulation testing results on goal-directed transfusion management could not be eliminated and should be taken into consideration. Third, we were using standard TEG to guide transfusion, rather than rapid TEG. Moreover, we were not able to perform “baseline TEG”, which was shown to be important for patients receiving TEG monitoring, since we were studying trauma patients in this study. Finally, this single institution experience may not be generalized because of different strategies in resuscitation, transfusion, and operation between trauma centers.

## Conclusions

In summary, the present study showed that goal-directed transfusion protocol via TEG was feasible in patients with abdominal trauma, and was better than conventional transfusion management in reducing blood product utilization and preventing coagulation function exacerbation. The results are in favor of implementation of goal-directed transfusion protocol in trauma patients. Further studies are needed to confirm the benefits of the novel transfusion strategy in the trauma setting.

## Abbreviations

VHA: Viscoelastic hemostatic assay; TEG: Thrombelastography; ED: Emergency department; SICU: Surgical intensive care unit; RBC: Red blood cell; FFP: Fresh frozen plasma; OR: Operation room; ISS: Injury severity score; AIS: Abdominal abbreviated injury scale; INR: International normalized ratio; aPTT: activated partial thromboplastin time.

## Competing interests

The authors declare that they have no competing interests.

## Authors’ contributions

JY and ZZ initiated the idea, carried out the study, and drafted the manuscript. JW, DY, and SZ helped collect and analyze data. YL and WY participated in the design of the study. NL and JL participated in the coordination of the study and helped to draft the manuscript. All authors read and approved the final manuscript.

## Authors’ information

Jianyi Yin and Zhenguo Zhao are joint first authors.
